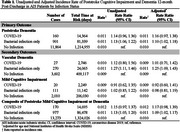# Long‐term Cognitive Impact of COVID‐19 Infection in Acute Ischemic Stroke Patients

**DOI:** 10.1002/alz70860_098693

**Published:** 2025-12-23

**Authors:** Hanzhang Xu, Brian C. Mac Grory, Uchechukwu Ikeaba, Brooke Alhanti, Gregg C. Fonarow, Lee H. Schwamm, Eric E. Smith, Steven R. Messe, Truls Ostbye, James A. De Lemos, Eric D. Peterson, Ying Xian

**Affiliations:** ^1^ Duke University, Durham, NC, USA; ^2^ Duke University School of Medicine, Durham, NC, USA; ^3^ University of California, Los Angeles, Los Angeles, CA, USA; ^4^ Yale University School of Medicine, New Haven, CT, USA; ^5^ Department of Clinical Neurosciences, University of Calgary, Calgary, AB, Canada; ^6^ Hotchkiss Brain Institute, University of Calgary, Calgary, AB, Canada; ^7^ University of Pennsylvania, Philadelphia, PA, USA; ^8^ UT Southwestern Medical Center ‐ Dallas, TX, Dallas, TX, USA; ^9^ UT Southwestern Medical Center, Dallas, TX, USA

## Abstract

**Background:**

About one in three COVID‐19 patients experience neurological problems; and acute ischemic stroke (AIS) is one of the common comorbidities of COVID‐19. As stroke doubles the risk of dementia, AIS patients with COVID‐19 infection may experience even higher risk of developing poststroke dementia. Still, little is known about the incidence of poststroke dementia in AIS patient with COVID‐19 infection.

**Method:**

Using data from the Get With The Guidelines–Stroke linked Medicare claims, we conducted a retrospective cohort study to estimate the incidence of poststroke dementia in AIS patients (1) with COVID‐19 with or without bacterial infection, (2) with bacterial infection only, and (3) with no infection. AIS Patients aged 66+ without pre‐stroke dementia from January 1, 2020, to June 30, 2021 were included and followed up for 12 months post discharge. Primary outcome—incident poststroke dementia—was obtained based on the International Classification of Diseases, Tenth Revision, from Medicare claims. Secondary outcomes include incident vascular dementia, poststroke mild cognitive impairment, and composite of poststroke mild cognitive impairment and dementia. Poisson regression was used to model the incidence of poststroke dementia accounting for age and stroke severity.

**Result:**

Of 103,378 patients (median [interquartile range] age, 79 [73‐86], 56,769 [54.9%] female; 84.074 [81.3.9%] white), 1,438 (1.4%) had COVID‐19 infection during stroke onset, and 6,084 (5.9%) had bacterial infection only. A total of 12,925 incident dementia cases developed during 1,310,658 person‐days after stroke onset. Poststroke dementia incidence rate was 0.011 in patients with COVID‐19 infection, 0.011 in patients with bacterial infection only, and 0.010 in patients with no infection. Compared to patients with no infection, the rates of poststroke dementia were significantly higher in patients with only bacterial infection (adjusted rate ratio[aRR]: 1.13 [95% CI, 1.06‐1.22]). Similar trends were observed in patients with COVID‐19 infection, although not statistically significant (aRR: 1.14 [95% CI, 0.96‐1.36]). Similar results were seen in the incidence of vascular dementia and composite cognitive outcome.

**Conclusion:**

We observed a higher incidence of poststroke dementia in AIS patients with bacterial infection only and those with COVID‐19 infection. COVID‐19 infection may be associated with higher risk of dementia post stroke.